# Evaluation of a drug-drug interaction: fax alert intervention program

**DOI:** 10.1186/1472-6947-13-32

**Published:** 2013-03-04

**Authors:** Edward P Armstrong, Sharon M Wang, Lisa E Hines, Sara Gao, Bimal V Patel, Daniel C Malone

**Affiliations:** 1 University of Arizona College of Pharmacy, 85721, Tucson, AZ, USA; 2 MedImpact Healthcare Systems, Inc., San Diego, California, USA

**Keywords:** Drug interactions, Drug safety, Physician, Prescriber, Fax

## Abstract

**Background:**

Clinicians often encounter information about drug-drug interactions (DDIs) during clinical practice. This information is found within product information (hardcopy and electronic) and various electronic systems. Prescribers may receive medication-related communications in practice that are distributed by facsimile (fax), mail, or telephone from pharmacies and pharmacy benefit managers (PBMs). The purpose of this study was to determine if near-real time fax alerts for potential drug-drug interactions (PDDIs) would influence prescribing.

**Methods:**

A prospective study, in cooperation with a pharmacy benefit manager (PBM), was conducted targeting 18 clinically important PDDIs. Fax alerts included an individualized letter to the prescriber with a list of the interacting drugs, PDDI evidence summaries with citations, and recommended clinical management strategies. Among the 18 PDDIs, 13 PDDIs could be assessed for prescription therapy changes using pharmacy claims data. A prospective cohort design was used to evaluate changes in prescription dispensing 90-days following a PDDI fax alert.

**Results:**

A total of 8,075 fax alerts were sent to prescribers and there were 4,712 alerts for the 13 PDDIs that could be assessed for change using pharmacy claims data. There were 2,019 patients (interventions) for which fax alerts were sent to their prescribers who were matched with a control group consisting of patients with the same PDDIs but for whom no fax alert was sent. Overall, this study found 154 (7.6%) of patients in the fax alert group compared to 132 (6.5%) in the control group had changes in therapy (p = 0.177).

**Conclusions:**

This fax alert intervention program observed no statistically significant differences in prescribing with a fax alert compared to the control group. If PBMs chose to send individualized, evidence-based information to clinicians regarding drug-drug interactions, this study suggests it may not be an effective intervention to mitigate harm.

## Background

Interventions to reduce harm associated with drug-drug interactions (DDIs) are commonly encountered by prescribers in everyday practice. These interventions are among many other medication-related communications directed to prescribers, including messages sent by facsimile (fax), mail, or telephone from pharmacies and pharmacy benefit managers (PBMs). Providers with electronic health records and/or electronic prescribing (e-prescribing) also are presented medication-related messages and alerts as a part of their systems’ clinical decision support. Because clinicians receive many alerts from a variety of sources, it is imperative to use only the most effective interventions aimed to improve patient safety related to DDIs.

PBMs are in a unique position to identify the co-prescribing of medications known to interact (i.e., potential drug-drug interactions or PDDIs) because they process and capture data on a large proportion of prescription claims in the United States [1]. PDDIs are detected through retrospective drug utilization review (DUR) programs that are conducted shortly after medications are dispensed to screen prescription claims data for patient exposure to specific DDIs. Letters may be sent to prescribers by fax or by mail to increase prescriber awareness or to request action, such as a change in therapy or increased monitoring [2]. However, some DUR programs have been implemented without satisfactory evidence to support the selected screening criteria or the effect of the interventions on prescribing patterns or patient outcomes [1-3]. To the authors’ knowledge, studies evaluating the effects of PDDI fax alerts on prescribing have not been published.

This study was planned and conducted in association with a PBM company (MedImpact Healthcare Systems, Inc.) to assess the impact of a fax alert program focused on select PDDIs. The purpose of this study was to measure the change in pharmacy claims after sending a fax alert to prescribers as compared to a control group that did not receive a fax alert. The study also evaluated prescribers’ opinions about the fax alert method but those results are reported elsewhere [4].

## Methods

This was a prospective cohort design study conducted to identify patients exposed to 18 PDDIs and to distribute evidence-based information and suggested actions (e.g., change drug, modify dosing or administration, monitoring, and/or patient education). Among the 18 PDDIs, 13 PDDIs could be assessed for prescription therapy changes using pharmacy claims data. Clinical information on the mechanism of action, potential severity of the interaction, recommended alternative medications and management strategies, and supporting references and documentation were included in the alert. The alerts were faxed the following morning (i.e., near-real time) to the prescriber of the second drug of the PDDI. A matched comparison (control) group, serving as a control where no fax alerts were sent, was selected from patients who were exposed to the same PDDIs identified using pharmacy claims from different health plans managed by the PBM. Changes in medication therapy for the 13 PDDIs were observed and compared among the intervention and control groups. A “successful change” was defined as initiation of a recommended therapeutic alternative within 90 days of the fax alert for the 13 PDDIs. The project was approved by the University of Arizona Human Subjects Protection Program.

### Interacting drugs combinations

Eighteen drug interactions considered to be clinically important were initially selected for the fax alerts by consensus among the authors (Table 1). These interactions were selected based on previous research [5]. The interactions of interest were refined based on input from the PBM, including a review of the potential clinical consequences and frequency of co-prescription [6]. An evidence-based summary was developed for each PDDI following a review of peer-reviewed publications, product labeling, drug compendia, and reputable online resources [7-12]. The most appropriate therapeutic options to minimize the risk of adverse reactions also were identified using primary (original research), secondary (databases of articles such as PubMed or Embase), and tertiary (review articles and book chapters) sources. Management options included in the alerts were: 1) continue treatment with patient education and follow-up, 2) modify the dose or frequency of administration, 3) monitor pharmacokinetic or pharmacodynamic parameters, 4) change to a therapeutic alternative that is less-likely to interact, or 5) temporarily hold, or discontinue therapy. However, claims analysis was not used to determine the potential impact of certain recommendations such as therapeutic drug monitoring, changing a dose or frequency, temporarily holding a medication, educating the patient, or asking the patient to monitor for certain signs or symptoms. Five of the 18 PDDIs (amiodarone-sotalol, ergotamines-triptans, nitrates-phosphodiesterase type 5 inhibitors, warfarin-amiodarone, and warfarin-thyroid) were sent for educational purposes to increase prescriber awareness, but were not evaluated for changes in prescribing. These five were considered to be clinically important PDDIs, but they could be managed by temporarily withholding a dose or through patient education to monitor for possible adverse events. These management strategies would not be detected by examining data submitted by pharmacies. Thus, a total of 13 PDDIs were included in this analysis (Table 1).

**Table 1:** Potential drug-drug interactions (PDDIs) and suggested therapeutic management strategies included in fax alert intervention program

**PDDIs**	**Suggested therapeutic management strategies**
**Amiodarone + Macrolides** (amiodarone + clarithromycin, erythromycin or azithromycin)	• Change antibiotic therapy: Change to non-macrolide or non-quinolone antibiotic
	*OR*
	• Monitor: Monitor ECG at baseline and periodically for QTc prolongation; educate patient about signs of symptomatic cardiac arrhythmias
**Amiodarone + Quinolones** (amiodarone + gemifloxacin, levofloxacin, moxifloxacin, or ofloxacin)	• Change antibiotic therapy: Change to ciprofloxacin, penicillins, cephalosporins; avoid changing to a macrolide (QTc prolongation)
	*OR*
	• Monitor: Monitor ECG at baseline and periodically for QTc prolongation; educate patient about signs of symptomatic cardiac arrhythmias
**Benzodiazepines + Azole Antifungal Agents** (alprazolam, midazolam, or triazolam + itraconazole, ketoconazole, fluconazole, posaconazole, or voriconazole)	• Change benzodiazepine therapy: Change to lorazepam, oxazepam, or temazepam
	*OR*
	• Change antifungal therapy: Change to terbinafine
	*OR*
	Monitor: Monitor for excessive sedation and prolonged hypnotic effects; counsel patients about these possible adverse effects; benzodiazepine dose reduction may be required
**Carbamazepine + Macrolides** (carbamazepine + erythromycin or clarithromycin)	• Change antibiotic therapy: Change to azithromycin or a non-macrolide antibiotic, such as 2nd/3rd generation cephalosporins, penicillins
	*OR*
	• Monitor: Monitor for changes in carbamazepine concentrations
**Ciprofloxacin + Tizanidine**	• Change antibiotic therapy: Change to alternative quinolone (e.g., gemifloxacin, levofloxacin, lomefloxacin, moxifloxacin, norfloxacin, ofloxacin) or non-quinolone antibiotic
	*OR*
	• Monitor: Monitor for evidence of tizanidine toxicity such as hypotension and excessive CNS depression
**Isotretinoin + Tetracycline or Minocycline**	Change antibiotic therapy: Change to a non-tetracycline antibiotic (e.g., macrolide, amoxicillin, sulfamethoxazole/ trimethoprim)
	*OR*
	• Monitor: Monitor and educate patient about signs and symptoms of pseudotumor cerebri (e.g., papilledema, headache, nausea, vomiting, visual disturbances)
**Simvastatin + Amiodarone**	• Change statin therapy: Change to fluvastatin, pravastatin, or rosuvastatin
	*OR*
	• Monitor: Monitor for unexplained muscle pain, tenderness, or weakness
**Statin + Azole Antifungal Agents** (atorvastatin, lovastatin, or simvastatin + itraconazole, ketoconazole, posaconazole, voriconazole, or fluconazole)	• Change statin therapy: Change atorvastatin, lovastatin, or simvastatin (when used in combination with itraconazole, ketoconazole, posaconazole, voriconazole, or fluconazole) to pravastatin OR change fluvastatin or rosuvastatin (when used in combination with fluconazole or voriconazole) to pravastatin, atorvastatin, lovastatin, or simvastatin
	*OR*
	• Modify: Temporarily stop the statin for short-term azole antifungal therapy or reduce the statin dosage
	*OR*
	• Monitor: Monitor for myopathy and possible rhabdomyolysis
**Statins + Macrolides** (atorvastatin, lovastatin, or simvastatin + erythromycin or clarithromycin)	• Change antibiotic therapy: Change to azithromycin or a non-macrolide antibiotic
	*OR*
	• Change statin therapy: Change to fluvastatin, pravastatin, or rosuvastatin
	*OR*
	• Temporarily stop the statin for short-term macrolide therapy
	*OR*
	• Monitor for signs of myopathy and possible rhabdomyolysis
**Theophylline + Quinolones** (theophylline + ciprofloxacin or norfloxacin)	• Change antibiotic therapy: Change to alternative quinolone (e.g., gemifloxacin, levofloxacin, lomefloxacin, moxifloxacin, ofloxacin) or non-quinolone antibiotic
	*OR*
	• Monitor: Monitor for changes in theophylline concentrations
**Warfarin + Azole Antifungal Agents** (warfarin + fluconazole, miconazole, or voriconazole)	• Change the antifungal agent: Change to itraconazole, ketoconazole, posaconazole, or terbinafine
	*OR*
	• Monitor: Monitor INR and for bleeding
**Warfarin + Fibrates** (warfarin + fenofibrate or gemfibrozil)	• Change lipid-lowering therapy: Change to pravastatin, fluvastatin, or possibly extended-release niacin or ezetimibe
	*OR*
	• Monitor: Monitor the INR and adjust the warfarin dosage as necessary
**Warfarin + Statins (**warfarin + fluvastatin, lovastatin, rosuvastatin, or simvastatin)	• Change statin therapy: Change to atorvastatin or pravastatin
	*OR*
	• Monitor: Monitor INR and for bleeding

CNS = central nervous system; ECG = electrocardiogram; INR = International Normalized Ratio.

### Data source

Two health plans participated in the study. One health plan was a large managed care organization and the other was a subset of a managed Medicaid health plan with approximately 500,000 and 20,000 covered lives, respectively. The PBM performed nightly scans of the plans’ pharmacy claims for detection of the select PDDIs and triggered a fax alert to the prescriber of the second interacting medication. The fax included a letter to the prescriber introducing the study, the patient’s name, a list of the interacting medications, and an evidence-based summary of the literature supporting the interaction. The managed care organization implemented the fax alert program for all 18 of the PDDIs. However, the Medicaid managed care plan did not implement alerts for the benzodiazepine-azole PDDI because of state regulatory concerns about behavioral health medications. The PDDI screening was based on specific medications and, therefore, individual prescribers received multiple fax alerts if they prescribed the same PDDI to multiple patients. In addition, if a single patient had more than one PDDI, the prescriber received additional fax alerts to address each PDDI. Prescriber specialties were identified for prescribers who received a fax alert based on Health Market Science data (updated January 2011) containing self-reported specialties. Because a practitioner may have multiple specialties, only the primary specialty listed was used for analysis.

The fax alert program was implemented for six months from May 2010 to November 2010. The control group consisted of patients for whom a PDDI also was identified by the same drug criteria but prescribers for these patients did not receive a fax alert. Control group patients were matched by PDDI type, although the drugs may have different active ingredients or formulations. A 1:1 case-control matching process was performed for each PDDI. Matching was based on age (± 2 years), gender, line of business (i.e., commercial managed care or managed Medicaid health plan) and time period the PDDI was detected. Usual pharmacist and physician interactions were allowed to occur without interference. Thirteen patient cohorts were constructed, one for each PDDI pair, and analyzed for changes in therapy. A patient could have been included in more than one cohort if the patient had multiple PDDIs. However, in the control group, a patient could only be matched to a single cohort even if they had multiple PDDIs.

The authors collectively determined the most appropriate management action based on available therapeutic alternatives that would be successful in limiting harm. Analysis of pharmacy claims was used to identify changes in therapy corresponding to recommendations provided in fax alerts for alternative therapies (see Table 1). New drug therapies, specified *a priori* as therapeutic alternatives that were filled within 90 days of when the fax alert was sent were defined as “successful changes” for the 13 of 18 PDDIs that could be evaluated.

Fax alert counts and changes in therapy were quantified for each PDDI pair. Chi square tests were conducted to determine if the proportion of successful therapy changes were significantly different between fax alert intervention and control groups. If the sample sizes were small (< 5 per group), a Fischer exact test was performed. A logistic regression model was created to assess the impact of variables that may influence a successful therapy change. The independent variable of interest was the fax alert intervention compared to the control group. Adjustment variables included: physician characteristics (specialty, type of health care practitioner), acute medication (i.e., drugs not considered as maintenance drugs by First DataBank that are commonly used for short term treatment), First DataBank drug interaction severity level, patient gender, patient age, and risk category of medication. RxRisk is a risk stratification model based on pharmacy claims data and was used as a proxy to identify disease comorbidities [13].

## Results

Of the total of 8,075 fax alerts distributed for all 18 PDDIs, 7,101 were sent for patients with at least 90 days of prescription plan eligibility after the fax alert. Of the 4,712 fax alerts sent for the 13 PDDIs evaluated for a change in prescribing, 2,019 could be successfully matched with a control.

Slightly greater than two-thirds of the patients (69.7%) with PDDIs were at least 65 years of age. Women represented 53.7% of PDDIs. The commercial managed care plan had 6,371 of 7,101 (89.7%) matched interventions. Physicians received 7,043 of 8,075 (87.2%) fax alerts sent and other health professionals received the balance of fax alerts. Prescribers categorized as primary care practitioners (defined as having a primary specialty of internal medicine, general practice, or family practice) received 3,764 of 8,075 (46.6%) fax alerts. The demographic characteristics of patients are summarized in Table 2.

**Table 2:** Patient demographics for intervention group

	**Aggregated**		**Client A**		**Client B**	
	**N**	**%**	**N**	**%**	**N**	**%**
Age						
<18	13	0.2	13	0.2		
18 to 39	135	1.9	67	1.1	68	9.3
40 to 64	2004	28.2	1368	21.5	636	87.1
> = 65	4949	69.7	4923	77.3	26	3.6
Gender						
Female	3813	53.7	3413	53.6	400	54.8
Male	3288	46.3	2958	46.4	330	45.2
LOB						
Commercial	6371	89.7	6371	100		
Medicaid	730	10.3			730	100
Total Fax Alerts	7101	100	6371	100	730	100

Comparison of “successful” changes between the fax alert intervention and control group are summarized in Table 3. For the 2,019 PDDI fax alerts matched to a control group, 154 (7.6%) patients had therapy changed in the fax alert group compared to 132 (6.5%) in the control group (p = 0.177). There was wide variation in the prescribing changes after the fax alert between the 13 different PDDIs. For example, in the statin-macrolide PDDI, there were 41 of 140 (29.3%) therapy changes after the fax alert compared to 42 of 140 (30.0%) changes in the control group (p = 0.89). For the warfarin-fibrate PDDI, there were 55 of 303 (18.2%) changes after the fax alert compared to 48 of 303 (15.8%) changes in the control group (p = 0.45). With the theophylline-quinolones PDDI, there were eight of 12 (66.7%) changes after the fax alert compared to two of 12 (16.7%) changes in the control group (p = 0.04).

**Table 3:** Counts of unique fax alerts sent and recommended therapy changes for intervention and control groups

***PDDI pair***	***Unique fax alerts sent***	***% of total PDDIs***	***Number of intervention group changes***	***Change rate (%)***	***Number of control group changes***	***Change rate (%)***	***p-Value***
Warfarin + Statins^a^	939	46.5%	9	1.0%	10	1.1%	0.82
Statins + Azoles^b^	247	12.2%	2	0.8%	2	0.8%	1.0
Warfarin + Fibrates^c^	303	15.0%	55	18.2%	48	15.8%	0.45
Simvastatin + Amiodarone	169	8.4%	8	4.7%	3	1.8%	0.13
Statins + Macrolides^d^	140	6.9%	41	29.3%	42	30.0%	0.89
Benzodiazepines + Azoles^e^	86	4.3%	9	10.5%	4	4.7%	0.15
Warfarin + Azoles^e^	62	3.1%	1	1.6%	1	1.6%	1.0
Ciprofloxacin + Tizanidine	24	1.2%	13	54.2%	11	45.8%	0.56
Isotretinoin + Tetracycline/Minocycline	15	0.7%	1	6.7%	1	6.7%	1.0
Amiodarone + Macrolides^f^	11	0.5%	2	18.2%	4	36.4%	0.63
Theophylline + Quinolones^g^	12	0.6%	8	66.7%	2	16.7%	0.04
Amiodarone + Quinolones^h^	7	0.3%	3	42.9%	2	28.6%	1.0
Carbamazepine + Macrolides^i^	4	0.2%	2	50.0%	2	50.0%	1.0
Total	2019	100.0%	154	7.6%	132	6.5%	0.177

NA = not applicable to calculate recommended therapy modification using a pharmacy claims database; PDE5 = phosphodiesterase type 5.

^a^ warfarin + atorvastatin, lovastatin, or simvastatin.

^b^ atorvastatin, lovastatin, or simvastatin + itraconazole, ketoconazole, posaconazole, voriconazole, or fluconazole.

^c^ warfarin + fenofibrate or gemfibrozil.

^d^ atorvastatin, lovastatin, or simvastatin + erythromycin or clarithromycin.

^e^ warfarin + fluconazole, miconazole, or voriconazole.

^f^ amiodarone + clarithromycin or erythromycin.

^g^ theophylline + ciprofloxacin or norfloxacin.

^h^ amiodarone + gemifloxacin, levofloxacin, moxifloxacin, or ofloxacin.

^i^ carbamazepine + erythromycin or clarithromycin.

As noted in Table 1, the recommended management strategy for the benzodiazepine-azole PDDI was to either change the benzodiazepine to lorazepam, oxazepam, or temazepam or instead to change the antifungal to terbinafine. There were eight patients where a change to lorazepam was noted, two changed to temazepam, and one changed to terbinafine. These therapy changes were consistent with the recommended management strategy suggested in the clinical information contained within the fax alert.

For the warfarin-fibrate PDDI, the suggested management strategy was to change therapy to pravastatin, fluvastatin, extended-release niacin, or ezetimibe and monitor the international normalized ratio (INR). There were 30 patients changed to ezetimibe with or without simvastatin, 28 patients were changed to pravastatin, 18 were changed to extended-release niacin, and 2 changed to fluvastatin. These therapy changes also were consistent with the recommended management strategy suggested in the fax alert.

For the ciprofloxacin-tizanidine PDDI, the preferred management strategy was to change the antibiotic to an alternative quinolone (e.g., gemifloxacin, levofloxacin, norfloxacin) or use a non-quinolone antibiotic. There were four patients where a change to levofloxacin was observed, three patients were changed to amoxicillin clavulanate, three were changed to cephalexin, two were changed to azithromycin, one was changed to amoxicillin, and one was changed to doxycycline.

For the simvastatin-amiodarone PDDI, the suggested management strategy was to change the statin to fluvastatin, pravastatin, or rosuvastatin. There were 10 patients that changed to pravastatin and one patient changed to rosuvastatin, as recommended by the fax alert.

For the warfarin-statin PDDI, the suggested management strategy was to change the statin to atorvastatin or pravastatin. There were 27 patients where the therapy was changed to pravastatin and eight where treatment was changed to atorvastatin.

The logistic regression analysis examined the relationship between successfully changing to a recommended therapy between the fax alert intervention group and the control group, adjusting for variables including prescriber characteristics, patient characteristics, and DDI characteristics (Table 4). The odds ratio of changes in therapy after the fax alert intervention was not found to be statistically different from the control group (p = 0.229).

**Table 4:** Logistic regression model for whether a patient’s PDDI resulted in a change in medication therapy

***Independent variable***	***Coefficient***	***P-value***	***Odds ratio***	***Odds ratio lower 95%***	***Odds ratio upper 95%***
Intercept	-1.9049	<.0001			
Intervention group	0.1706	0.229	1.186	0.898	1.566
Specialist	-0.1179	0.4246	0.889	0.666	1.187
Practitioner Type					
Nurse Practitioner	0.0993	0.7384	1.104	0.617	1.978
Physician’s assistant	-0.726	0.0729	0.484	0.219	1.07
Other Professional	-0.6397	0.3211	0.527	0.149	1.866
Physician (Reference)					
Acute Medication	0.1962	0.3247	1.217	0.823	1.798
FDB^*^ Severity Level					
1-major	0.4161	0.141	1.516	0.871	2.638
2-moderate (Reference)					
3-minor	-2.7735	<.0001	0.062	0.044	0.089
Male Patient	0.3722	0.0137	1.451	1.079	1.951
Patient’s Age Group					
0 - 49	0.1565	0.5175	1.169	0.728	1.878
50 - 64	0.0455	0.7896	1.047	0.749	1.462
65 - 79 (Reference)					
80 and Up	0.0877	0.6948	1.092	0.705	1.691
Patient’s RxRisk Categories [13]					
Anxiety & Tension	0.3507	0.0343	1.42	1.026	1.965
Asthma	0.3336	0.2724	1.396	0.769	2.533
Cardiac Disease	0.2865	0.1145	1.332	0.933	1.901
Coronary/ Peripheral Vascular disease	-0.5916	0.0015	0.553	0.384	0.798
Depression	0.4939	0.0011	1.639	1.217	2.207
Diabetes	-0.107	0.4921	0.898	0.662	1.219
Epilepsy	0.0764	0.6746	1.079	0.755	1.542
End stage renal disease	0.0499	0.7402	1.051	0.783	1.412
Glaucoma	-0.2878	0.4534	0.75	0.353	1.591
Heart Disease/ Hypertension	-0.1678	0.2887	0.846	0.62	1.153
Hyperlipidemia	0.3231	0.1391	1.381	0.9	2.12
Irritable Bowel Syndrome	0.0359	0.8678	1.037	0.68	1.581

^*^First DataBank.

## Discussion

This was a prospective cohort design study that assessed changes in prescribing after sending patient-specific, evidence-based information about PDDIs with recommended management strategies to prescribers via fax. Overall, change in treatment was demonstrated in the pharmacy claims within 90 days of sending the fax alert packets in 7.6% of patients in the fax alert group. The only significant finding was that for prescribers who received the fax alert for theophylline-quinolones PDDIs, patients were more likely to have a pharmacy claim within 90 days for a non-interacting alternative than those patients in the control group. As measured, there was no evidence within pharmacy claims that the intervention had an effect on the use of alternative therapies for any of the other PDDIs. There are several possible explanations for these findings. First, prescribers may have already been aware of these PDDIs and had taken their potential risk into account. They also may have made changes in therapy that differed from those suggested in the fax alert packet and these prescribing changes were therefore not detected. Since new exposure to PDDIs was not specifically targeted, patients may have previously been exposed to and tolerated the medication combination prior to the prescriber receiving the alert. Moreover, prescribers may not have perceived the evidence-based review as compelling enough to change treatment. Using pharmacy claims data to measure the effect of the program may not fully account for the true program effect. Physicians, after receiving these alerts, may have limited prescribing of future PDDIs, thereby leading to a potential sentinel effect. Lastly, prescribers may not have reviewed the fax alert; for instance, the fax may have been intercepted by office staff and discarded. Unlike DDI alerts in e-prescribing, there is no way to determine what proportion of fax alerts were received and reviewed by prescribers.

The PDDIs for this fax alert intervention were selected based on the potential clinical consequences, frequency of co-prescription, and the scientific evidence supporting the interactions. Unfortunately, the evidence for DDIs is generally lacking, making “evidence-based” recommendations a challenge [14]. The majority of DDI studies consist of case reports and premarketing studies evaluating nonclinical endpoints in healthy subjects. Research also has shown inconsistencies in DDI listings and severity ratings among various information sources [15-17]. In hindsight, other PDDIs might have been more appropriate for targeted intervention to observe significant changes in prescribing. For example, the large number of warfarin PDDIs may have been managed within anticoagulation clinics. However, an up-to-date list of high-priority DDIs for alerting is a moving target and requires careful selection, validation, and ongoing evaluation.

Retrospective DUR programs that communicate drug-related problems to prescribers by fax or mail are common in managed care and some studies indicate these interventions may change prescribing behavior [18-24]. For example, Starner et al reported that nearly half of potentially inappropriate prescription medications in older adults were discontinued within six months of a retrospective DUR invention involving an information packet mailed to prescribers of one or more drugs to avoid in the elderly [24]. In contrast, Bambauer et al. found that fax alerts to prescribers about possible nonadherence among patients with late refills for antidepressants did not improve refill rates over two years after the fax intervention [25]. The authors suggested that faxed feedback should be carefully evaluated before widespread use because when used alone, it was inadequate. Unlike the present study, few studies that evaluate the effects of retrospective DUR incorporate a control group [18,23].

The role of faxes has also been evaluated in other health care interventions. Rosewell et al. studied physicians that had received a fax regarding a measles outbreak and noted that recipients were more likely to be aware of the measles epidemic [26]. The respondents indicated the measles fax alert was useful. Chen et al. conducted a blinded, randomized, controlled trial that examined the effectiveness of delivering computer-generated discharge summaries to general practitioners by email, fax, mail, or patient hand delivery [27]. Receipt of the discharge summary was similar with email and fax, and both of these were significantly higher than mail or patient hand delivery. Practitioners indicated that fax was the preferred method.

At first glance, drug safety notifications alerting prescribers makes intuitive sense. However, Weingart et al. estimated that a small proportion (10%) of automated drug safety alerts likely accounted for preventing the majority (60%) of the adverse drug events [28]. Studies report high rates (>90%) of alert override in e-prescribing systems and overall dissatisfaction with drug safety alerts [29-31]. One survey of physicians found that respondents were generally unsatisfied with DDI and allergy alerts, based on issues such as outdated information, failure to account for appropriate drug combinations, and excessive alert volume [31]. Peng et al. noted that the incidence of potentially serious DDIs was less than 1% of prescriptions in ambulatory patients [20]. They encouraged the use of additional “automated filters” when screening pharmacy claims for PDDIs with “additional pharmacist review” [20].

Challenges of retrospective DUR programs include questionable clinical relevance of certain DDIs, poor evidence for many interactions, inconsistencies among knowledge bases and compendia, and incomplete patient data. Those managing and using DUR should consider criteria that screen for significant preventable problems [32]. Criteria should include PDDIs with potentially severe medical consequences if action is not taken, strong evidence to support an association (pharmacokinetic or pharmacodynamic) with the outcome of interest, and be logically and practically feasible to manage the PDDI with available data and then be able to track the change [2]. An expert consensus may be useful to identify the most important DDIs from a systematic review of the clinical literature, although this process should aim for acceptable levels of sensitivity and specificity and provide a framework for local adaptation [1,33]. Furthermore, a critical need remains for evaluating the effects of DDIs on patient outcomes.

With advances in clinical decision support systems such as integration of relevant medical and laboratory information, and national attention focused on meeting meaningful use criteria, DDI alert programs will continue to evolve toward more electronic means for communications. Even so, the same issues about the need for careful selection and evaluation of screening criteria apply to electronic DDI alerts. The absence of careful, informed assessment of knowledge databases used for DDI screening can lead to alerting for PDDIs that are theoretical, unfounded, exaggerated or erroneous [34,35]. In addition, minimizing PDDIs is becoming more important for PBMs and health plans in the US because Medicare is now using quality metrics that include the incidence of PDDIs.

There are several important limitations with this study. Many PDDIs may not lend themselves to being assessed using a pharmacy claims database since these data are not useful to detect increased physician awareness and patient monitoring, patient education, or temporarily holding of a chronic medication. Examining changes in claims data to measure the effect of the program may not fully account for the full program effect as prescribers may alter future prescribing after receiving an alert. Additionally, after receiving these alerts, physicians may have limited prescribing of future PDDIs, thereby leading to a potential sentinel effect. In addition, specialists are presumably more knowledgeable about certain therapies (e.g., dermatologists and isotretinoin-minocycline or tetracycline or doxycycline) and these may have been less useful PDDIs to target with a fax alert intervention. For example, isotretinoin prescribers need to be certified to prescribe the drug. The large number of warfarin PDDIs may have been managed within anticoagulation clinics with extra monitoring. Next, sample sizes for select PDDI types were small and not all patients with PDDIs were matched to a comparator, which reduced the sample size and power of the statistical analysis. Also, because specific prescribers could have received multiple fax alerts for the same or other interactions, they may have been less responsive to the PDDI fax alert. The study was not designed to collect the number of fax alerts received by a prescriber. In addition, this study was “near-real time” in design and responded when new interacting prescriptions were written. It was unknown whether the interacting drug pair had been previously used in the patient’s history.

It also should be noted that because the fax alert program was near-real time, it was possible for prescribers to intercept prescriptions before the patient picked them up. If that was the case, then the claim would have been reversed (or voided) and would not be captured as it examined prescription claims data. Also, pharmacies are typically equipped with software that provides PDDI screening and many PBMs provide soft messaging warnings when claims are adjudicated. The usual clinical care and communications that occur between prescribers and pharmacists may have resolved the PDDI issues by other processes not observable in the pharmacy claims data.

## Conclusion

Based on the results from this fax alert analysis, there were no significant changes in prescription medications obtained by patients between the intervention and control prescribers. While using solely claims-based analyses for this evaluation resulted in no detectable differences, if PBMs elect to use fax alerts to inform clinicians about clinically relevant PDDIs, it is likely not to have an observable impact on the claims utilization of therapeutic alternatives. Future research should examine if prescribers used other harm mitigation strategies for patients exposed to PDDIs.

## Abbreviations

DDIs: Drug-drug interactions;DUR: Drug utilization review;Fax: Facsimile;INR: International normalized ratio;PBMs: Pharmacy benefit managers;PDDIs: Potential drug-drug interactions

## Competing interests

Author Disclosure: Drs. Armstrong, Malone, and Hines have nothing to declare. Drs. Wang and Patel and Ms. Gao are employees of MedImpact Healthcare Systems, Inc.

## Authors’ contributions

EA, LH, and DM were involved in the conception, design, and writing of the paper. SW, SG, and BP were involved revising the study methods, conducting the analysis, and revising the manuscript. All authors read and approved the final manuscript.

## Pre-publication history

The pre-publication history for this paper can be accessed here:

http://www.biomedcentral.com/1472-6947/13/32/prepub
